# Links Between Personality and Sleep Midpoint in Older Adults in the National Social Life, Health, and Aging Project

**DOI:** 10.1093/geroni/igab046.128

**Published:** 2021-12-17

**Authors:** Krishna Patel, Darlynn Rojo-Wissar, Katherine Duggan, Garrett Hisler, Brant Hasler, Adam Spira

**Affiliations:** 1 Penn State College of Medicine, Hummelstown, Pennsylvania, United States; 2 Department of Mental Health, Johns Hopkins Bloomberg School of Public Health, Baltimore, Maryland, United States; 3 Department of Psychology, North Dakota State University, Fargo, North Dakota, United States; 4 Department of Psychiatry, University of Pittsburgh, Pittsburgh, Pennsylvania, United States; 5 Johns Hopkins Bloomberg School of Public Health, Baltimore, Maryland, United States

## Abstract

Chronotype has been linked to poor cognitive outcomes and mortality among older adults. Although previous studies indicate an association between personality and sleep, little is known about associations between personality and chronotype in older adults. We examined the association between personality and objective sleep midpoint (a measure of chronotype) in 463 older adults aged 73.5 ±7.7 from the National Social Life, Health, and Aging Project who completed the Midlife Developmental Inventory Personality scale and three nights of wrist actigraphy, from which we derived participants’ average sleep midpoints. After adjusting for demographics, higher conscientiousness was associated with earlier sleep midpoint (B=-0.53, SE=0.02, p<0.01). Associations for other traits were not significant. Findings link conscientiousness to chronotype and raise the possibility that earlier sleep timing may partially account for associations of conscientiousness with health outcomes. Further studies are needed investigating the role of personality in links of sleep and circadian factors with health.

